# New Formulation of a Methylseleno-Aspirin Analog with Anticancer Activity Towards Colon Cancer

**DOI:** 10.3390/ijms21239017

**Published:** 2020-11-27

**Authors:** Ana Carolina Ruberte, Gustavo González-Gaitano, Arun K. Sharma, Carlos Aydillo, Ignacio Encío, Carmen Sanmartín, Daniel Plano

**Affiliations:** 1 Department of Pharmaceutical Technology and Chemistry, University of Navarra, Irunlarrea 1, E-31008 Pamplona, Spain; aruberte@alumni.unav.es (A.C.R.); caydillo@unav.es (C.A.); 2 Instituto de Investigación Sanitaria de Navarra (IdiSNA), Irunlarrea 3, E-31008 Pamplona, Spain; ignacio.encio@unavarra.es; 3 Department of Chemistry, University of Navarra, 31080 Pamplona, Spain; gaitano@unav.es; 4 Department of Pharmacology, Penn State Cancer Institute, CH72, Penn State College of Medicine, Hershey, PA 17036, USA; asharma1@pennstatehealth.psu.edu; 5 Department of Health Sciences, Public University of Navarra, Avda. Barañain s/n, E-31008 Pamplona, Spain

**Keywords:** aspirin, cytotoxicity, colorectal cancer, cyclodextrins, Pluronic, selenium

## Abstract

Aspirin (ASA) has attracted wide interest of numerous scientists worldwide thanks to its chemopreventive and chemotherapeutic effects, particularly in colorectal cancer (CRC). Incorporation of selenium (Se) atom into ASA has greatly increased their anti-tumoral efficacy in CRC compared with the organic counterparts without the Se functionality, such as the promising antitumoral methylseleno-ASA analog (**1a**). Nevertheless, the efficacy of compound **1a** in cancer cells is compromised due to its poor solubility and volatile nature. Thus, **1a** has been formulated with native α-, β- and γ-cyclodextrin (CD), a modified β-CD (hydroxypropyl β-CD, HP-β-CD) and Pluronic F127, all of them non-toxic, biodegradable and FDA approved. Water solubility of **1a** is enhanced with β- and HP- β-CDs and Pluronic F127. Compound **1a** forms inclusion complexes with the CDs and was incorporated in the hydrophobic core of the F127 micelles. Herein, we evaluated the cytotoxic potential of **1a**, alone or formulated with β- and HP- β-CDs or Pluronic F127, against CRC cells. Remarkably, **1a** formulations demonstrated more sustained antitumoral activity toward CRC cells. Hence, β-CD, HP-β-CD and Pluronic F127 might be excellent vehicles to improve pharmacological properties of organoselenium compounds with solubility issues and volatile nature.

## 1. Introduction

Aspirin (ASA) is an extensively used drug for a variety of indications. Over the past decade, ASA has attracted attention of numerous scientists worldwide owing to its promising chemopreventive and chemotherapeutic effects, particularly against colorectal cancer (CRC) [1]. Several clinical studies have shown that low dose of ASA prevents CRC and reduces its incidence and risk of metastasis [2,3]. However, the long-term use of ASA is associated with severe gastrointestinal side effects. Thus, numerous efforts have been made to optimize ASA in order to improve its efficacy [4,5,6,7,8,9].

**Figure 1:**
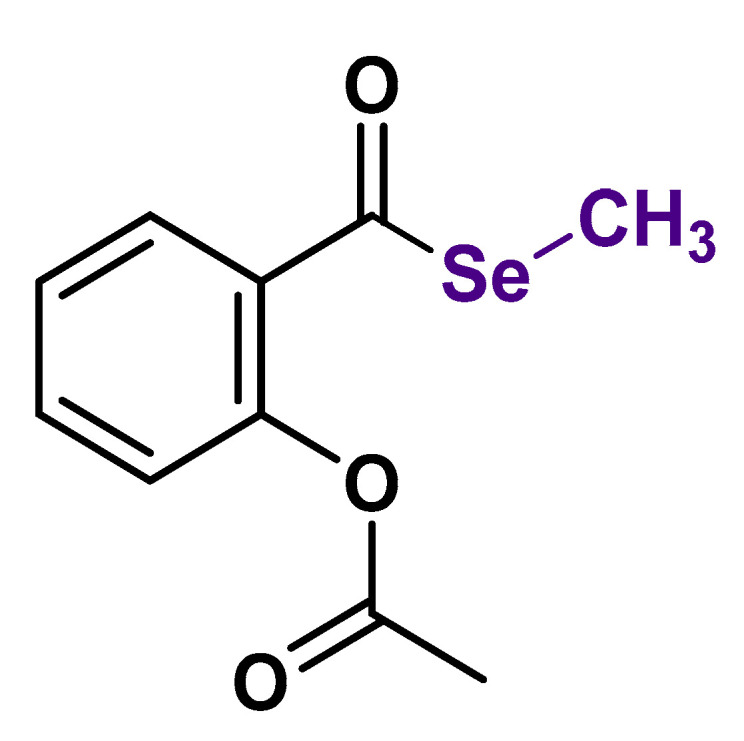
Structure of methylseleno-aspirin (ASA) analog (2-([methylselanyl]carbonyl)phenyl acetate, **1a**).

On the other hand, incorporation of selenium (Se) into nonsteroidal anti-inflammatory drugs (NSAIDs) has demonstrated to tremendously increase their anti-tumoral efficacy compared with the organic counterparts without the Se functionality [7,10,11,12]. Remarkably, the antitumoral efficacy of Se depends on the rate of its metabolic conversion to mono-methylated Se species, such as methylselenol (CH_3_SeH). This is one of the most significant metabolites responsible for the antitumoral activity of Se [13]. Thus, numerous CH_3_SeH precursor have been designed and synthesized, such as methylseleninic acid, which demonstrated inhibitory efficacy toward several cancers, particularly CRC [14]. Using these principles and following a fragment-based design, our research group identified a promising antitumoral methylseleno-ASA analog (2-([methylselanyl]carbonyl)phenyl acetate, **1a**), which is a putative active methylselenol (CH_3_SeH) precursor (Figure 1). This promising antitumoral agent was synthesized in one-pot by reaction of NaHSe with *O*-acetylsalicyloyl chloride and the subsequent intermediate was then methylated with iodomethane [15]. Compound **1a** demonstrated a mean cell growth inhibition value of 54.63% against 60 cell lines, as was determined by the National Cancer Institute’s (NCI) human tumor cell lines screen. Additionally, in our laboratory derivative **1a** dissolved in DMSO, exhibited IC_50_ values below 2 μM in CRC [15], but DMSO cannot be used as a vehicle in a clinical setting. However, its water solubility is minimal and this analog also suffers from loss efficacy in cancer cells due to its volatile nature. Hence, **1a** require the need to develop clinically relevant formulations without compromising its efficacy.

To further develop this analog, we propose to resolve its limiting barriers through vehiculation techniques, which would increase its water solubility and make its extended-release form, hence enhancing its bioavailability and effectiveness on cancer cells. In this context, cyclodextrins (CDs) are one of the most used vehiculation methods for small molecules [16,17]. CDs are cyclic oligosaccharides formed by 6, 7, or 8 glucose units (α-, β- or γ-CD, respectively) linked by α-1,4 glycosidic bonds making a natural molecular cone-shaped container, with a slightly hydrophobic cavity and a hydrophilic exterior part. Thus, CDs can form a host–guest inclusion complex with hydrophobic drugs through non-covalent interactions, improving the water solubility and stability of drugs [18]. Remarkably, several pre-clinical studies confirmed that these inclusion complexes can also improve antitumoral drugs’ efficacy [16,17,19,20].

On the other hand, polymeric micelles are also emerging as encouraging nanocarriers to improve intracellular drug accumulation and drug efficacy. Pluronic^®^ family of surfactants (BASF) are linear triblock copolymers, in which two hydrophilic poly ethylene oxide (PEO) blocks are connected via a hydrophobic poly propylene oxide (PPO) block. Their amphiphilic properties, are markedly dependent on the number of EO or PO monomers [21,22]. They exhibit inhibitory activity toward overexpression of drug efflux transporters of the ATP-binding cassette [21,23]. Furthermore, some preclinical studies have proven that these constructs can enhance chemotherapeutic drugs’ efficacy, such as doxorrubicin [24].

Hence, **1a** has been formulated with different type of CDs (β-CD, α-CD, 2-hydroxypropyl-β-CD (HP-β-CD) and γ-CD) and Pluronic F127, an amphiphilic block copolymer that forms core-shell micelles. These drug constructs were characterized by NMR and UV–vis spectroscopies. We evaluated the cytotoxic potential of **1a** toward CRC, both alone and encapsulated with CDs or Pluronic F127. Likewise, we studied the in vitro capacity of these vehiculation techniques of holding and releasing the volatiles active fragment, which could be CH_3_SeH. Briefly, native β-CD, modified HP-β-CD and Pluronic F127 have been shown to be excellent vehicles to improve pharmacological properties of **1a**.

## 2. Results and Discussion

### 2.1. Water Solubility of **1a**

Compound **1a** showed poor water solubility (2.20 × 10^−4^ M) as determined by ^1^H-NMR (Figures S2 and S3). Thus, we have formulated **1a** with native CDs (α-CD, β-CD and γ-CD) and a modified HP-β-CD at room temperature, and Pluronic F127 (1% *w/v*) at 37 °C. Among all the CDs tested, only β-CD and HP-β-CD improved the solubility of the drug (Figures S4–S6), up to 6-fold (1.37 × 10^−3^ M and 1.46 × 10^−3^ M, respectively, at a CD concentration of 4.15 × 10^−3^ M and 4.17 × 10^−3^ M, respectively). Similarly, Pluronic F127 also increased the solubility (1.17 × 10^−3^ M, at 1% of surfactant).

### 2.2. Characterization of the Formulations

#### 2.2.1. Characterization of **1a**:CDs Complexes

**Figure 2:**
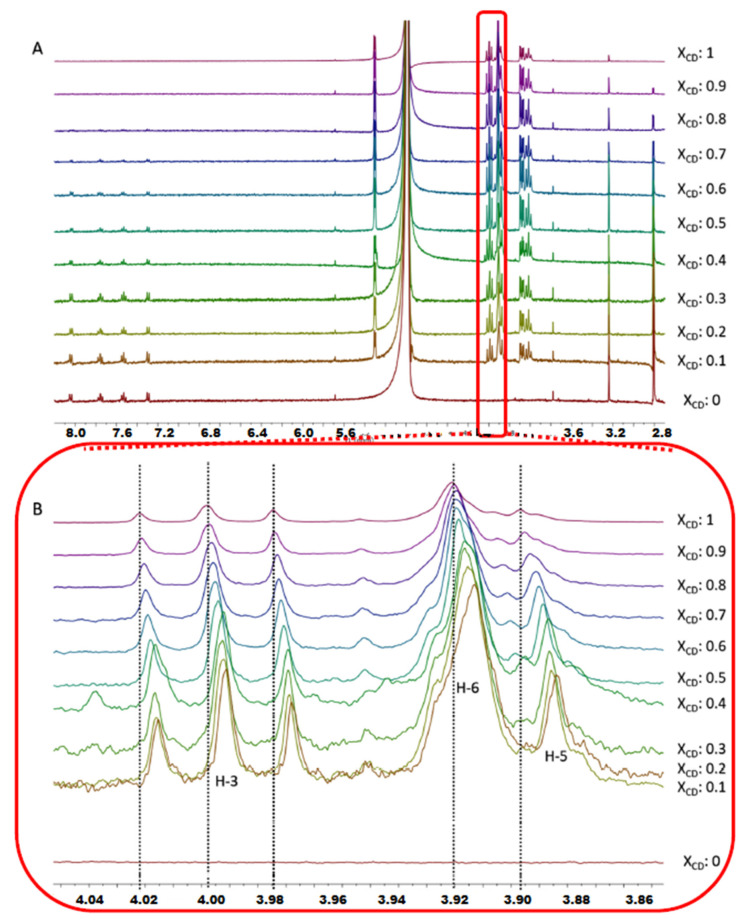
(**A**) ^1^H-NMR spectra for different molar fractions of **1a**:β-CD; (**B**) expansion of the region of **H-3**, **H-5** and **H-6** protons of β-CD.

**Figure 3:**
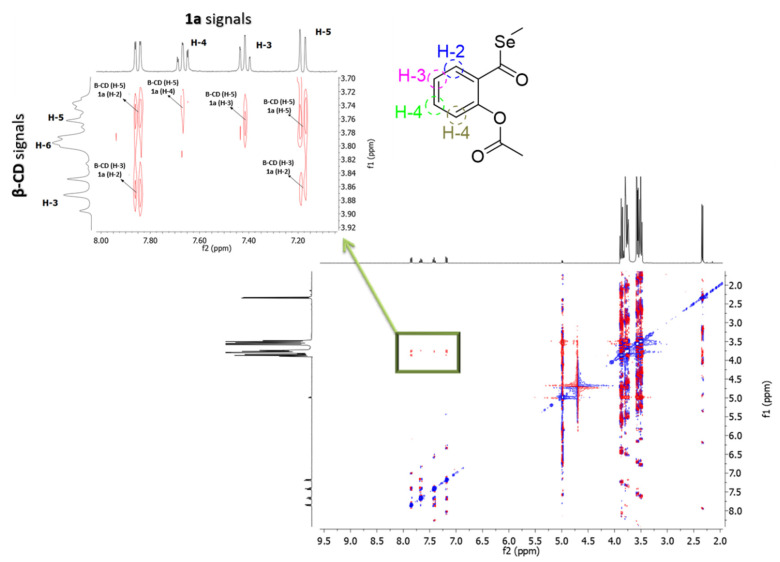
2D-ROESY-NMR spectrum of **1a**:β-CDs complex (1:1 molar ratio).

**Figure 4:**
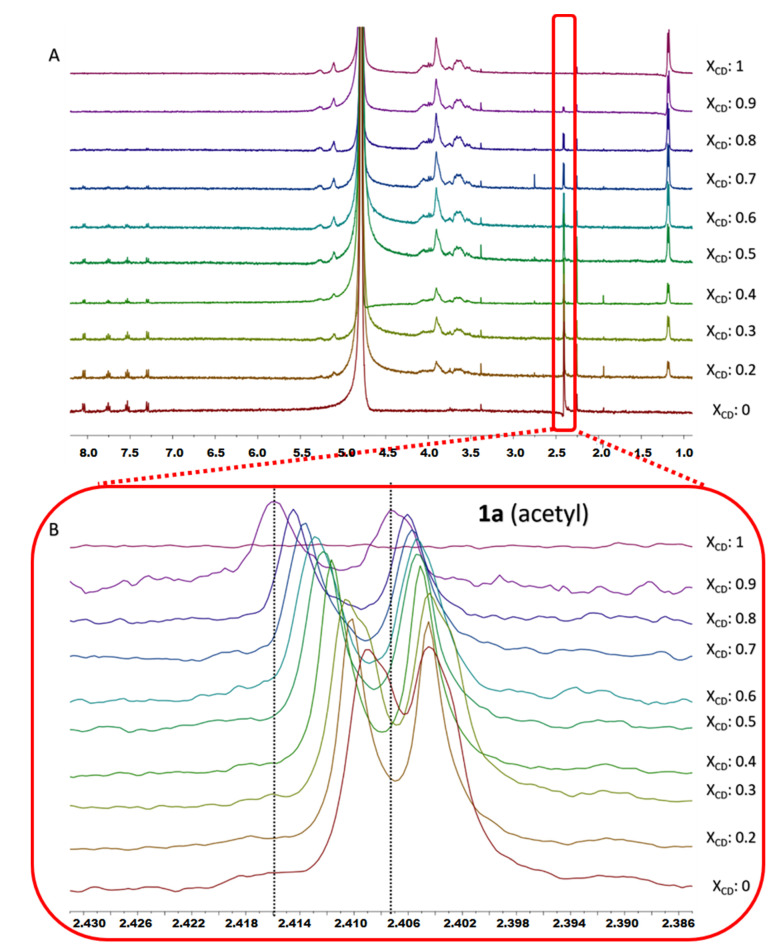
(**A**) ^1^H-NMR spectra of different molar fractions of **1a**:HP-β-CD and (**B**) expansion of the region of the acetyl protons of **1a**.

The formation of inclusion complexes of **1a** with the CDs was confirmed by ^1^H-NMR spectroscopy, given that inclusion of a guest in the CD always produces some changes in the chemical environment of the protons of the cavity. Specifically, the addition of **1a** to β-CD in D_2_O led to downfield shifts on **H-5** protons consistent with the inclusion of the drug in the CD cavity (Figure 2A,B). This inclusion complex was also confirmed by the ROESY-NMR spectrum, which showed strong cross-peaks between the inner **H-3** and **H-5** protons of β-CD and **H-2**, **H-4**, **H-3** and **H-5** aromatic protons of **1a** (Figure 3). These results prove unambiguously that the aromatic ring of **1a** is localized inside the cavity of the macrocycle. The inclusion complex of **1a** with the HP-β-CD was characterized by ^1^H-NMR with different molar fractions of **1a**:HP-β-CD in D_2_O (Figure 4A,B). The obtained spectra showed an upfield variation of the chemical shift of acetyl protons of **1a**. This finding confirms the binding between acetyl protons of **1a** and HP-β-CD, making this interaction very different compared with the one observed for **1a**:β-CD complex.

**Figure 5:**
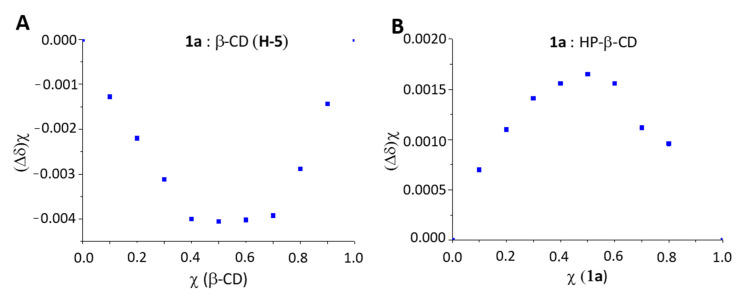
Job’s plot for **1a**:β-CD and **1a**:HP-β-CD complexes, for (**A**) **H-5** proton of β-CD and (**B**) acetyl protons of **1a,** respectively.

The Job’s plot method was applied using the proton shifts to ascertain the stoichiometry [25], which resulted in 1:1 stoichiometry with both CDs, according to the molar ratio at which the curve peaks at *r* = 0.5 (Figure 5A,B, respectively).

**Figure 6:**
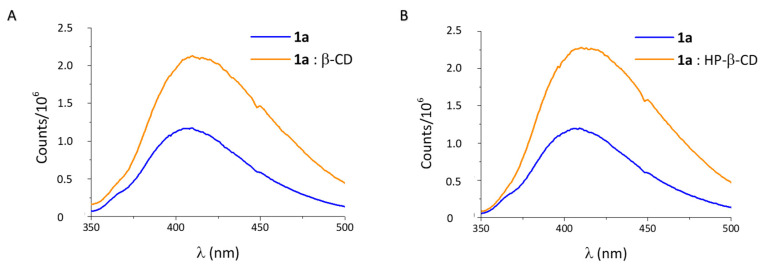
Fluorescence emission spectra of (**A**) **1a**:β-CD (1:30 molar ratio) and (**B**) **1a**:HP-β-CD (1:50 molar ratio). **1a** concentration is 2.2 × 10^−4^ M.

Inclusion complexes can also deeply change the physicochemical features of the guest molecule, like their absorbance in the UV–vis spectrum or its fluorescence emission [26]. In this case, the fluorescence spectrum of **1a** (2.2 × 10^−4^ M) was recorded in the absence and presence of the CDs. With any of both macrocycles the fluorescence emission of **1a** was remarkably increased (Figure 6A,B), along with a redshift of the band (ca. 10 nm), confirming the change in the micropolarity of the environment of **1a** as the results of the inclusion in the CD cavity.

**Figure 7:**
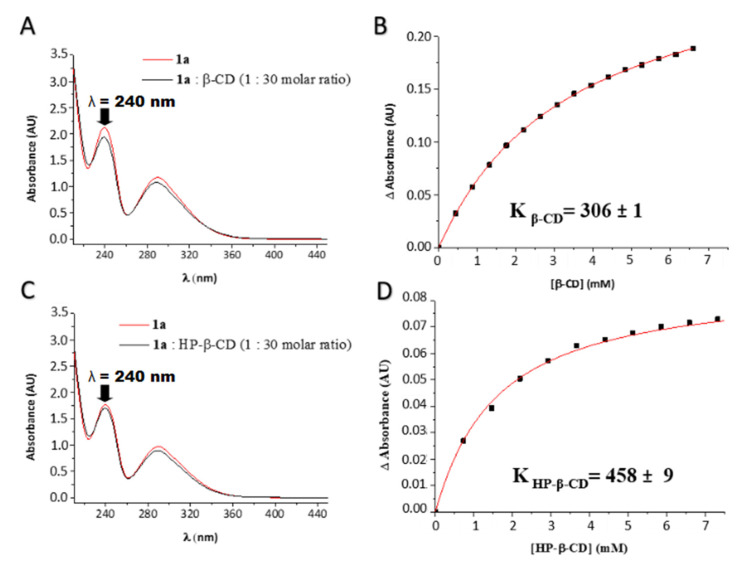
(**A**,**C**) Absorption spectra of 2.2 × 10^−4^ M of **1a** in the presence of β-CD and HP-β-CD (6.6 × 10^−3^ M), respectively. (**B**,**D**) Change in the maximum of absorbance at 240 nm (ΔA) as a function of the CD concentration.

We also studied the UV absorption spectra of **1a** (2.2 × 10^−4^ M) with increasing concentrations of β-CD and HP-β-CD (up to 1:30 molar ratio). Spectra showed small but significant differences in the maximum of absorbance at 240 nm when varying the concentration of the oligosaccharides (Figure 7A,C). These changes can be used to determine the binding constants, K_b_, of the complexes **1a**:CDs [27], from the non-linear regression of the ΔA data at 240 nm as a function of the concentration of CD (Figure 7B,D). The association constants of **1a**:β-CD and **1a**:HP-β-CD complexes were 306 ± 1 and 458 ± 9, respectively, showing a somewhat higher affinity of HP-β-CD for **1a**, compared to β-CD.

#### 2.2.2. Effects of Pluronic F127 Micelles on **1a**

**Figure 8:**
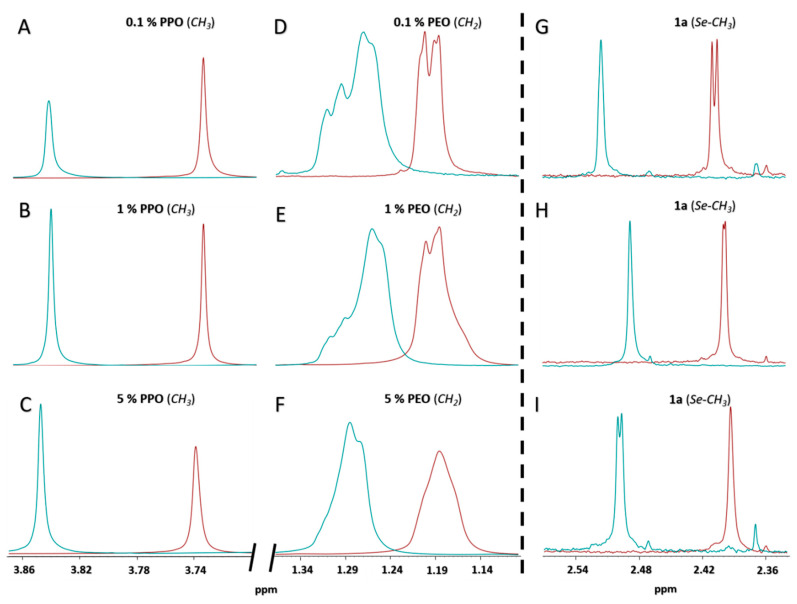
Expanded ^1^ H-NMR spectra of Pluronic F127 solutions (0.1%, 1% and 5% (*w/v*)) showing the signal of methyl (CH_3_) groups of the PPO (**A**–**C**) and methylene (CH_2_) groups of the PEO (**D**–**F**). Expanded ^1^ H-NMR spectra of **1a** in presence of 0.1%, 1% and 5% (*w/v*) Pluronic F127 solutions showing methylseleno (Se-CH_3_) group signal for **1a** (**G**–**I**, respectively), at 27 °C (red lines) and 37 °C (blue lines).

Polymeric micelles based on PPO and PEO block copolymers are strongly influenced by the temperature and the concentration, with implications in the loading capacity of the surfactant of poorly water-soluble drugs [28]. In this sense, NMR is a very suitable technique to investigate self-aggregation phenomena and the solubilization of small molecules in the micelles [29]. Figure 8 contains an expansion of the ^1^H-NMR spectra of F127 in D_2_O at different concentrations, at 27 and 37 °C, in the absence and presence of **1a**. The most relevant signals of the copolymer are the doublet at 3.73 ppm of the methyl groups of the PPO block (Figure 8A–C) and the intense singlet peak at 1.20 ppm, corresponding to the protons of the methylene groups of the PEO block (Figure 8D–F). Focusing first on the surfactant without **1a**, and regarding the temperature effect, both groups of signals shift upfield upon heating (0.11 ppm the methyl protons, Figure 8A–C, and 0.06 the methylene protons, Figure 8D–F, when passing from 27 to 37 °C). This shift can be interpreted in terms of the dehydration of the blocks that occurs when the unimers self-aggregate to form the micelles, which involves changes in the magnetic environment of the corresponding protons. This dehydration is more remarkable for the PPO blocks, which form the core of the micelle, due to its higher hydrophobicity compared with the PEO. Regarding the concentration effect at a given temperature, the micellization of Pluronic F127 occurs in a higher extent with 5% (*w/v*), while less concentration of the surfactant is needed, 1% (*w/v*) to have the micelles formed at 37 °C (Figure 8B,E, blue lines).

**Figure 9:**
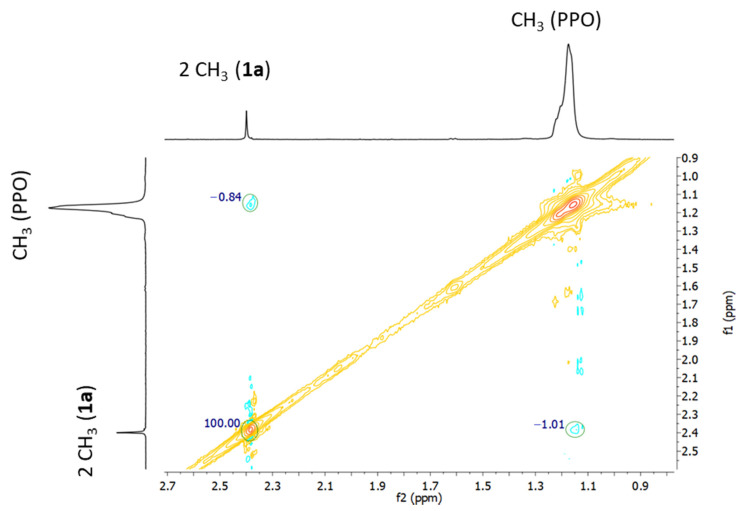
Expansion of the NOESY-NMR spectrum of complex **1a**:F127 (1% (*w/v*)) at 37 °C.

In the presence of drug, changes in the resonances of **1a** reflect the different magnetic environments. The expanded ^1^ H-NMR spectrum of **1a** with 1% (*w/v*) F127 at 37 °C (micelles fully formed) showed an intense singlet peak at 2.5 ppm, assigned to the protons of the methylseleno (Se-CH_3_) group of **1a** (Figure 8H, blue line). Additionally, aromatic signals of 1a under these same conditions are gathered on Figure S7. Upon increasing the concentration of surfactant the resonances shift upfield, confirming the interactions between the polymer and the drug. The cross-peak between the methyl protons of the PO monomers and both methyl groups of **1a** (methylseleno and acetyl) observed in the NOESY spectrum confirm that the drug is located mainly in the hydrophobic core (Figure 9), which justifies the increase in the solubility of **1a** in water, when it is formulated with the copolymer.

### 2.3. Antitumoral Activity against CRC Cells

**Figure 10:**
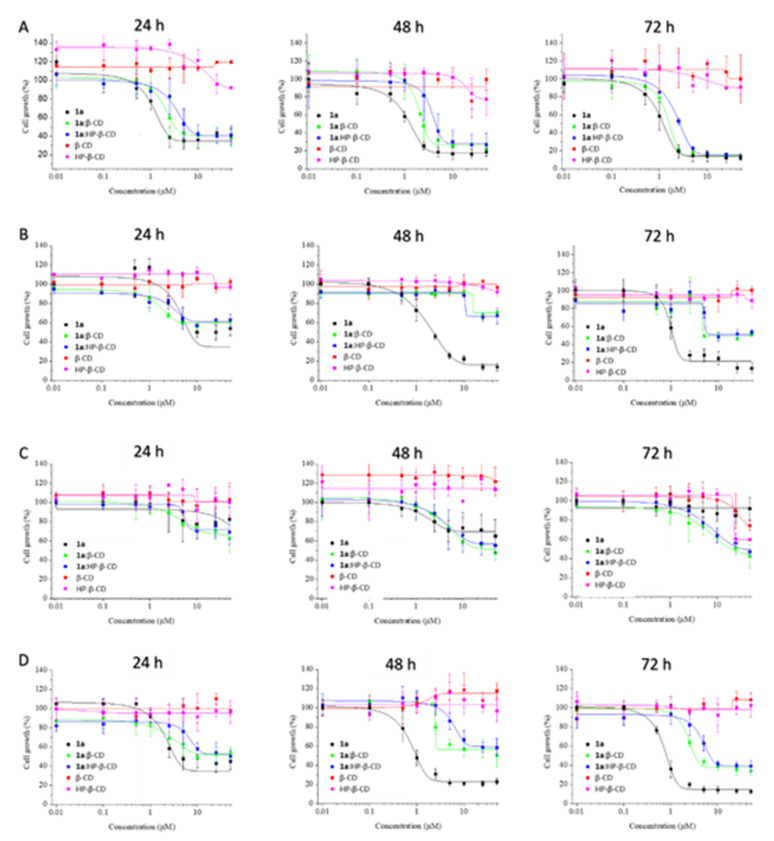
Dose-response curves obtained for **1a** (black lines), β-CD (red lines), HP-β-CD (pink lines), as well as the formulations of **1a** with β-CD (green lines) and **1a** with HP-β-CD (blue lines) on HT-29 (**A**), HCT-116 (**B**), RKO (**C**) and Caco-2 (**D**) cell lines at 24, 48 and 72 h.

**Figure 11:**
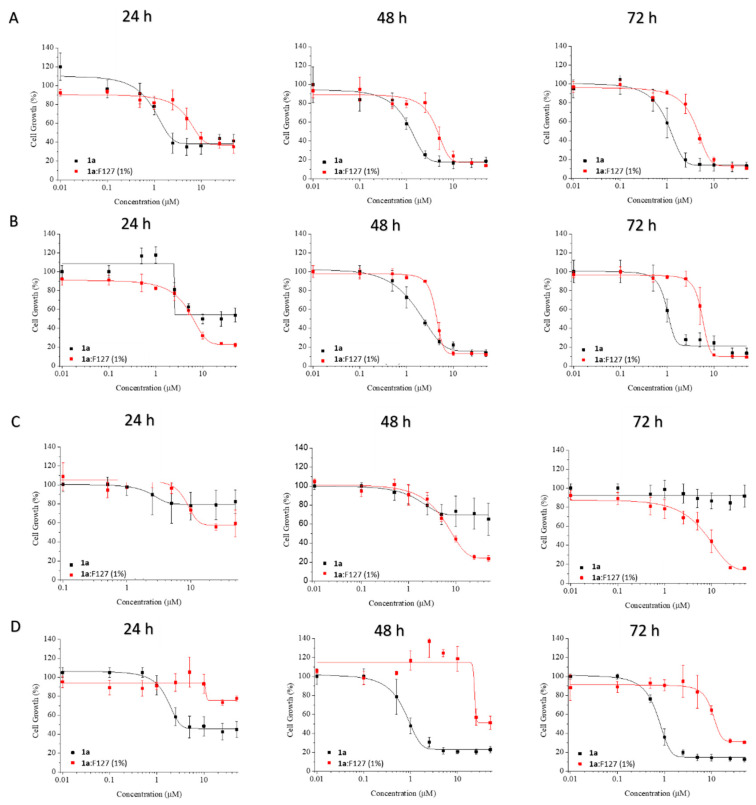
Dose-response curves obtained for **1a** (black lines) and **1a**:F127 (red lines), on HT-29 (**A**), HCT-116 (**B**), RKO (**C**) and Caco-2 (**D**) cells at 24, 48 and 72 h.

**1a**, dissolved in DMSO, has proven effective on CRC cells [15]. However, DMSO cannot be used as a vehicle in a clinical setting and the water solubility of **1a** is minimal. So, the drug constructs, dissolved in water, were evaluated in vitro toward CRC cells (HT-29, HCT-166, RKO, Caco-2) at 24, 48 and 72 h using MTT assay [30]. The CRC cells used were chosen in order to cover different features including sensitivity to 5-fluorouracil and oxaliplatin (HT-29), wild-type p53 expression (RKO), as well as expression of transforming growth factor beta-1 and -2 (HCT-116) and retinoic acid binding protein I and retinol binding protein II (Caco-2). Dose-response curves for each cell line and time point are depicted in Figure 10 and Figure 11. Regarding RKO cells, the cytotoxic potential of **1a** improved when forming CD inclusion complexes or when incorporated to F127 polymeric micelles, even at 24 h. Further research is needed in order to find an explanation to this unexpected outcome considering that RKO is the only CRC cell line showing this behavior. It might be because this cell line is the less differentiated one among the CRC cells evaluated. On the contrary, for the rest of CRC cell lines (HT-29, HCT-116 and Caco-2), the cytotoxic potential of **1a** decreased at 24 h, although the cytotoxic activity was recovered at 72 h. This fact can be explained by a slower release of **1a** or some active volatiles, such as methylselenol, when formulated with CDs or F127. Hence, the vehiculation of the drug by using CDs or dissolved in a polymeric micelle could prolong its cytotoxic activity in the tumor to increase the effectiveness of **1a** analog. Remarkably, the complex **1a**:β-CD displayed greater cytotoxic activity than the complex with HP-β-CD, which agrees with the higher affinity constant with HP-β-CD. Finally, HT-29 cell line was the most sensitive CRC cell line and was selected for studying the qualitative release of possible active volatiles for **1a** itself [15].

### 2.4. Release Studies

**Figure 12:**
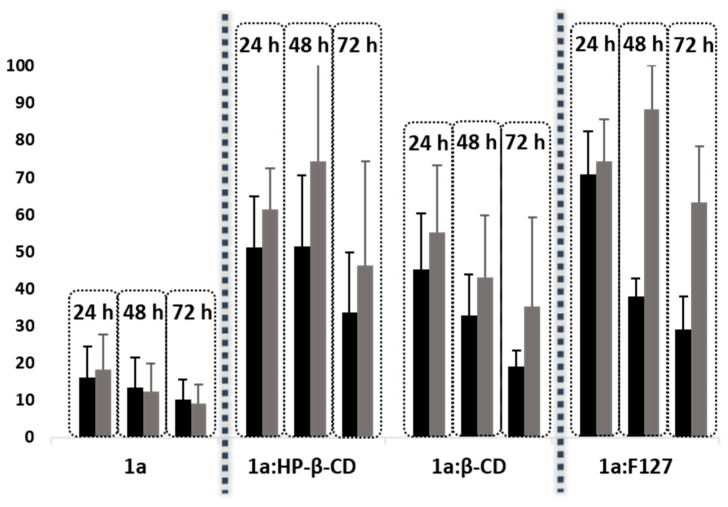
Cell growth of HT-29 cells treated with vehicle (DMSO) located adjacent to the cells treated with 50 (Control A, black) or 25 µM (Control B, grey) of **1a**, **1a**:HP-β-CD, **1a**:β-CD or **1a**:F127 after 24, 48 and 72 h.

The release of volatile components of **1a** alone or formulated was qualitatively evaluated in vitro toward HT-29 cell line at three times (24, 48 and 72 h) and three concentrations (50, 25 and 10 µM) using the MTT assay (Figure S1) [30]. Results are expressed as percent of cell growth (Figure 12). Interestingly, **1a** alone displayed high cytotoxic activity toward HT-29 cells on adjacent well to the treatments. This observation confirmed that **1a** is precursor of active volatile components. Remarkably, when the cells were treated with **1a** formulated with CDs or Pluronic F127, its cytotoxic potential toward cells on adjacent wells decreased, suggesting a reduction of active volatile fragments release (Figure 12). The release of volatiles for the complex **1a**:β-CD was higher than with **1a**:HP-β-CD, probably due to its lower affinity constant (Figure 12). The lowest cytotoxicity activity toward cells in adjacent wells was exhibited by **1a**:F127.

## 3. Discussion

In the literature, numerous NSAID-releasing derivatives have been developed and characterized. Thus, phospho-, HS-, NO- and NOSH-releasing NSAIDs have demonstrated potent cytotoxic activity toward different cancer cells [31,32,33,34,35,36]. Considering only aspirin, NO- [34,37,38] and phospho-releasing [6] analogs stand out as antitumor agents against CRC. Notwithstanding, compound **1a** is the first Se-aspirin releasing agent, showing IC_50_ values ranging from 0.9 to 2.2 µM on HT-29, HCT-116 and Caco-2 CRC cell lines [15].

Few examples of NSAIDs containing Se have been reported [7,11,12,39,40], all of them showing a tremendous increase of antitumor activity compared with the non-modified NSAIDs. Even though all of them show IC_50_ values comparable with compound **1a**, this new Se-aspirin analog possesses a great advantage given its ability to affect untreated cells by releasing active volatiles. This key feature could represent a massive advantage for treating not only primary tumors but also distant metastatic lesions, a concept that remains to be tested. Potentially, treatment of tumors with compound **1a** could affect not only cells on the tumor surface but also cells on the inner portion of the tumor. However, this feature could also represent the main drawback for the use of this compound as it can also be cleaved before reaching the tumor.

The translational fate of any small molecule depends on how efficiently it can be encapsulated in a clinically relevant formulation. Therefore, to advance compound **1a** to pre-clinical in vivo evaluation as a prelude to possible clinical studies in future, we designed several formulations aiming to achieve more sustained release of active volatiles with the treatment. Previously, our research group used Pluronics and Tetronics to increase water-solubility of very hydrophobic selenodiazoles, with optimal results [41]. Nevertheless, to our knowledge this is the first time that a methylseleno-releasing compound is formulated with cyclodextrins and Pluronics. The new formulated methylseleno-aspirin analog presents greater features than parent compound **1a** since cytotoxic activity is sustained overtime, thus improving its chances to succeed in a possible clinical evaluation. Although this study is only on preliminary stages of development and extensive research is still needed, we consider these formulations have the potential to be clinically developed.

Numerous clinical studies have been conducted with cyclodextrins [42,43] and Pluronics F127 [44], showing both vehicles are adequate for clinical development. Accordingly, we propose for an eventual future clinical development of these formulations the following groups for comparison: (1) cyclodextrins, Pluronics and **1a** alone, (2) examples of phospho-, H_2_S- and NO-releasing aspirin and, (3) methylseleninic acid, as a methylselenol precursor.

## 4. Materials and Methods

### 4.1. Materials

**1a** was synthesized as previously reported [15]. Native α-CD (≥98%), β-CD (≥97%), and γ-CD (≥98%), with water contents of 10%, 14%, and 10% respectively, were obtained from Sigma-Aldrich (MO, USA). A modified HP-β-CD (0.8 molar substitution), Pluronic copolymer F127, comprising a central block of 65 PPO units and two side-blocks of PEO (100 units each), and diclofenac sodium salt were also obtained from Sigma-Aldrich.

### 4.2. Nuclear Magnetic Resonance (NMR) Studies

A Bruker Avance NEO 400 spectrometer (9.36 T) was used for recording the proton spectra of **1a** and its mixtures with the CDs and Pluronic in D_2_O (≥99.85% in deuterated component). To ascertain the stoichiometry of the complexes with the CDs, one-dimensional (1D) proton spectra were recorded for a series of solutions with molar ratios of the drug ranging from 0 to 1. Two-dimensional (2D)-ROESY and NOESY experiments were performed with the CDs and Pluronic, respectively, to probe the intermolecular interactions drug-carrier and the preferential location of the drug. For the 2D-ROESY, a 200 ms spin-lock mixing time was introduced in the pulse sequence. Solvent suppression was applied in all cases and the experiments run at 298 K (complexes with CDs) and 310 K (**1a** with F127)

### 4.3. Water Solubility Studies

Solubility studies of **1a** and complexes of **1a** with the CDs were carried out adding an excess of **1a** (2 mg) to a 0.5 mL D_2_O solution. This solution contained 1.75 mg of diclofenac sodium salt (internal standard) and different CDs (β-CD, α-CD, HP-β-CD and γ-CD) at 10 mM. Suspensions were maintained under stirring at room temperature for 30 min. Then, they were filtered and the supernatant analyzed by ^1^H-NMR (Figures S2–S4). Quantitative determination by this method is based on the premise that the integrated signal is proportional to the molar concentration of the substance that generates the resonance [45]. The solubilities have been calculated considering the area of the hydrogens of the diclofenac sodium salt (aryl hydrogens), β-CD (hydrogen 1), HP-β-CD (hydrogen 1) and Pluronic F127 (methylene hydrogens), which integrate for 1, 7, 7 and 198 protons per molecule, respectively. In the case of the **1a**:F127 formulation, the solubility was determined by adding an excess of **1a** (2 mg) to a 0.5 mL D_2_O solution containing 1% *w/v* of Pluronic F127. Suspension was stirred at 37 °C for 30 min and the solution then filtered and analyzed at the same temperature by ^1^H-NMR (Figure S5).

### 4.4. Characterization of the **1a**:CDs Inclusion Complexes

#### Stoichiometry Determination Using Job´s Method

Different molar fractions (0, 0.1, 0.2, 0.3, 0.4, 0.5, 0.6, 0.7, 0.8, 0.9, 1) of the complexes of **1a** with β-CD and HP-β-CD were prepared and the ^1^H-NMR spectra in D_2_O collected. The total concentration of both host and guest was kept constant at 0.4 mM, and the molar fractions *χ* of each component was varied from zero to one [25].

### 4.5. Binding Constant Determination of the Inclusion Complex by UV–Vis Spectroscopy

UV–vis absorption spectra were recorded on an Agilent 8453 UV–vis diode array spectrophotometer (200−800 nm; Agilent Technologies, Waldbronn, Germany), using 1 cm path-length quartz cuvettes. Changes in the absorption intensity of 1a (λ = 240 nm) were monitored as a function of β-CD or HP- β-CD concentration to determine the binding constant (K_b_), which was calculated by non-linear squares fitting with OriginPro 8.5.1 (Northampton, MA, USA). In these experiments the concentration of **1a** was kept constant at 2.2 × 10^−4^ M, whereas the concentration of β-CD and HP-β-CD varied from 0 to 6.9 × 10^−3^ M (1:30 molar ratio) by direct titration with a micropipette on the measuring cell.

#### Fluorescence Measurements

The binding of **1a** with β-CD or HP-β-CD was also studied using the intrinsic fluorescence in water at 21 °C. The fluorescence emission spectra of **1a** (2.2 × 10^−4^ M) and the complex of **1a** with β-CD (1:30 molar ratio) or HP-β-CD (1:50 molar ratio) were recorded in an Edinburgh Instruments FLS920 spectrofluorometer (Livingston, UK) using a 1 cm path length quartz cell. The excitation was set to 325 nm and the emission scanned from 340 to 500 nm at 1 nm steps and 0.1 s dwell time, with excitation and emission slits of 3 and 8 nm, respectively.

### 4.6. Characterization of the Solubilization of **1a** in F127 Micelles

The solubilization of **1a** in Pluronic F127 micelles was characterized by ^1^H-NMR. Pluronic F127 samples 0.1%, 1% and 5% *w/v* were prepared in D_2_O in the presence and absence of **1a** (2 mg/mL) at 27 and 37 °C. All samples were allowed to equilibrate at the desired temperature for 30 min prior to the measurements in the spectrometer.

### 4.7. Cell Culture Conditions

The cell lines were obtained from the American Type Culture Collection (ATCC). Caco-2 cell line was maintained in DMEM medium (Gibco-BRL, Gaithersburg, MD); and HT-29, HCT-116 and RKO cells were maintained in McCoy’s 5A medium (Gibco-BRL, Gaithersburg, MD), supplemented with 10% fetal bovine serum (FBS; Gibco-BRL, Gaithersburg, MD) and 1% antibiotics (10.00 units/mL penicillin and 10.00 mg/mL streptomycin; Gibco-BRL, Gaithersburg, MD). Cells were preserved in tissue culture flasks at 37 °C and 5% CO_2_. Culture medium was replaced every three days.

### 4.8. Cell Viability Assay

**1a** as single agent or encapsulated with CDs or Pluronic F127 were evaluated in vitro toward different CRC cell lines (HT-29, HCT-116, RKO and Caco-2) using the MTT assay [30]. First, 3000 cells/well were grown in 96-well plates for 12 h. Second, cells were incubated with either DMSO (control) or nine different concentration between 0.01–50 µM of **1a**, β-CD and HP-β-CD and their respectives inclusion complexes (1:1 molar ratio), for 24, 48, and 72 h. Regarding to polymeric micelles, cells were incubated with 1% of Pluronic F127 (control) or nine different concentration between 0.01–50 µM of **1a** in presence of 1% of F127, for 24, 48, and 72 h. Third, 20 µL of MTT were added to measure cellular viability. Finally, the resulting formazan crystals were dissolved in 50 µL of DMSO, and absorbance was measured at 570 nm and 630 nm wavelengths. Dose response curves were calculated using OriginPro 8.5.1 (Northampton, MA, USA).

### 4.9. Volatiles Active Release Studies

Volatiles active release by drug constructs were qualitatively evaluated in vitro against HT-29 cell line at three time points (24, 48 and 72 h) and four concentrations (100, 50, 25 and 10 µM), using MTT assay [30]. Four controls with DMSO were used. Three of them located adjacent to the cells treated with 100, 50 or 25 µM of **1a** and drug constructs (**1a**:β-CD or **1a**:HP-β-CD (1:1 molar ration) inclusion complexes or **1a** with 1% of F127). The results are expressed as percent of cell growth.

## 5. Conclusions

In summary, given the promising antitumoral activity of the methylseleno-aspirin analog **1a** but its loss of efficacy due to its scarce solubility and volatile nature, this analog was formulated with native α-, β- and γ-CD and a modified β-CD (HP-β-CD). The solubility of **1a** is enhanced, up to 6-fold, with β- and HP-β-CD and Pluronic F127, due to the formation of inclusion complexes with the β- and HP- β-CDs in a 1:1 stoichiometry, with binding constants (K_b_) of 306 and 458, respectively. Additionally, **1a** was also formulated with polymeric micelles of Pluronic F127, which also increases the solubility, incorporating the drug to the hydrophobic core of the micelles. In both cases the antitumoral effect of **1a** toward CRC cell lines was more sustained, probably as a consequence of an increase of its solubility and a slower release and the reduction in the release of active volatiles out of the cell. Native β-CD, modified HP-β-CD and Pluronic F127 can thus be promising vehicles to improve the pharmacological properties of organoselenium compounds, which usually present solubility and efficacy issues.

## Supplementary Materials

The following are available online at https://www.mdpi.com/1422-0067/21/23/9017/s1. Figure S1. Cell growth of HT-29 cells after treatments with **1a**, **1a**:HP-β-CD, **1a**:β-CD and **1a**:F127 at 50 µM (green), 25 µM (grey) and 10 µM (blue) after 24, 48 and 72 h. Cells treated with vehicle (DMSO) located adjacent to the cells treated with 50 µM (Control A, orange) or 25 µM (Control B, purple) of **1a** or supramolecular structures, were used as controls. Figure S2. ^1^H-NMR of **1a**. Figure S3. ^1^H-NMR of **1a** and diclofenac sodium salt. Figure S4. ^1^H-NMR of **1a** and β-CD. Figure S5. ^1^H-NMR of **1a** and HP-β-CD. Figure S6. ^1^H-NMR of **1a** and F127, at 37 °C. Figure S7. Expanded ^1^ H-NMR spectra of **1a** in the presence of Pluronic F127 (0.1%, 1% and 5% (*w/v*)) showing the signal of aryl protons of **1a** (**A**–**C**, respectively) at 27 °C (red lines) and 37 °C (blue lines).

## Abbreviations

ASA	Aspirin
CH_3_SeH	Methylselenol
CRC	Colorectal cancer
CDs	Cyclodextrins
HP-β-CD	2-Hydroxypropyl-β-cyclodextrin
MTT	3-(4,5-dimethylthiazol-2-yl)-2,5-diphenyltetrazolium bromide
NCI	National Cancer Institute
NMR	Nuclear magnetic resonance
NSAIDs	Nonsteroidal anti-inflammatory drugs
PEO	Poly ethylene oxide
PPO	Poly propylene oxide
Se	Selenium
