# Supplementary material for: New Formulation of a Methylseleno-Aspirin Analog with Anticancer Activity Towards Colon Cancer
Source: Int J Mol Sci. 2020 Nov 27;21(23):9017. doi: 10.3390/ijms21239017 (PMC7730823; doi:10.3390/ijms21239017)
Supplement: Supplementary file 1 [file ijms-21-09017-s001.pdf]

New Formulation of a Methylseleno-Aspirin Analog with anticancer activity towards colon cancer

Ana Carolina Ruberte, [1,2] *Gustavo González-Gaitano,* [3] *Arun K. Sharma,* [4] *Carlos Aydillo,* [1,2] *Ignacio Encío,* [2,5] *Carmen Sanmartín* [1,2,*] *and Daniel Plano* [1,2,*]

[1] Department of Pharmaceutical Technology and Chemistry, University of Navarra, Irunlarrea 1, E-31008 Pamplona, Spain.

[2] Instituto de Investigación Sanitaria de Navarra (IdiSNA), Irunlarrea 3, E-31008 Pamplona, Spain.

[3] Department of Chemistry, University of Navarra, 31080, Pamplona, Spain.

[4] Department of Pharmacology; Penn State Cancer Institute, CH72; Penn State College of Medicinal, Hershey PA 17036, USA

[5] Department of Health Sciences, Public University of Navarra, Avda. Barañain s/n, E-31008 Pamplona, Spain.

*E-mail: sanmartin@ unav.es, Tel.: +34 948 425600; E-mail: dplano@unav.es, Tel.: + 34 948 425600.

Table of Contents

**Release studies**	....	**Figure S1**
**NMR spectra**		
**1a**	....	**Figure S2**
**1a + diclofenac sodium**	....	**Figure S3**
**salt**	....	**Figure S4**
**1a + β-CD**	....	**Figure S5**
**1a + HP-β-CD**	....	**Figure S6**
**1a + F127**	....	**Figure S7**
**1a + F127 (0.1, 1 and 5% (w/v)**		

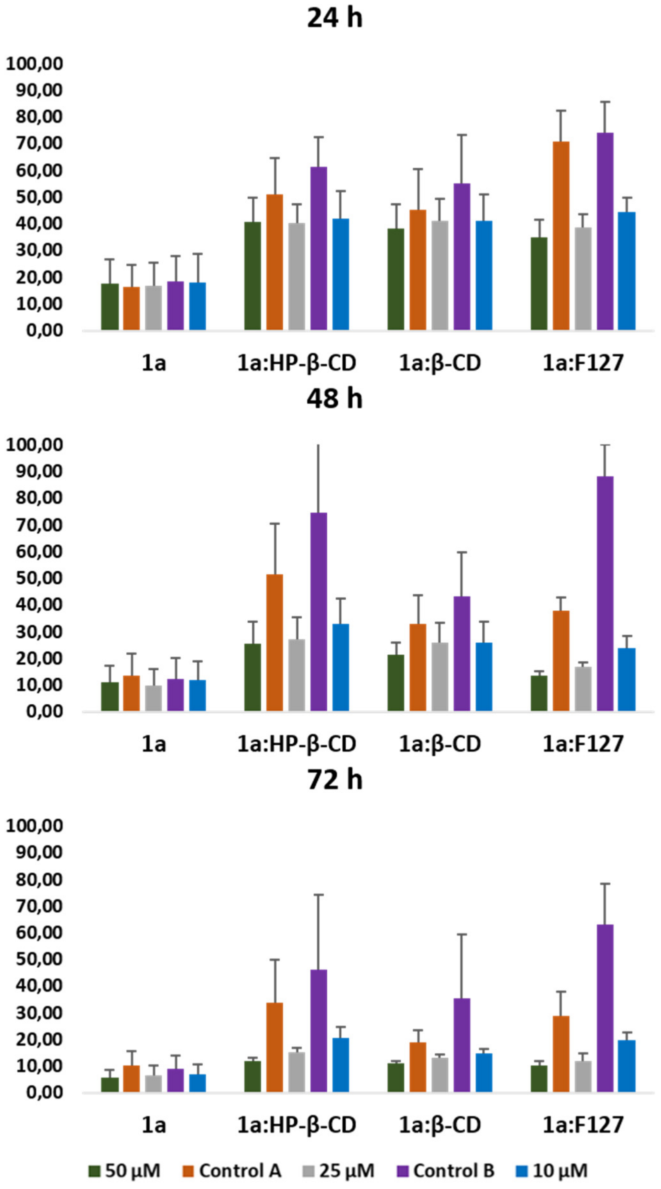

Figure S1. Cell growth of HT-19 cells after treatments with **1a**, **1a** : HP-β-CD, **1a** : β-CD and **1a** : F127 at 50 (green), 25 (grey) and 10 µM (blue) after 24, 48 and 72 h. Besides, cells treated with vehicle (DMSO) located adjacent to the cells treated with 50 (Control A, orange) or 25 µM (Control B, purple) of **1a** or supramolecular structures, were used as controls.

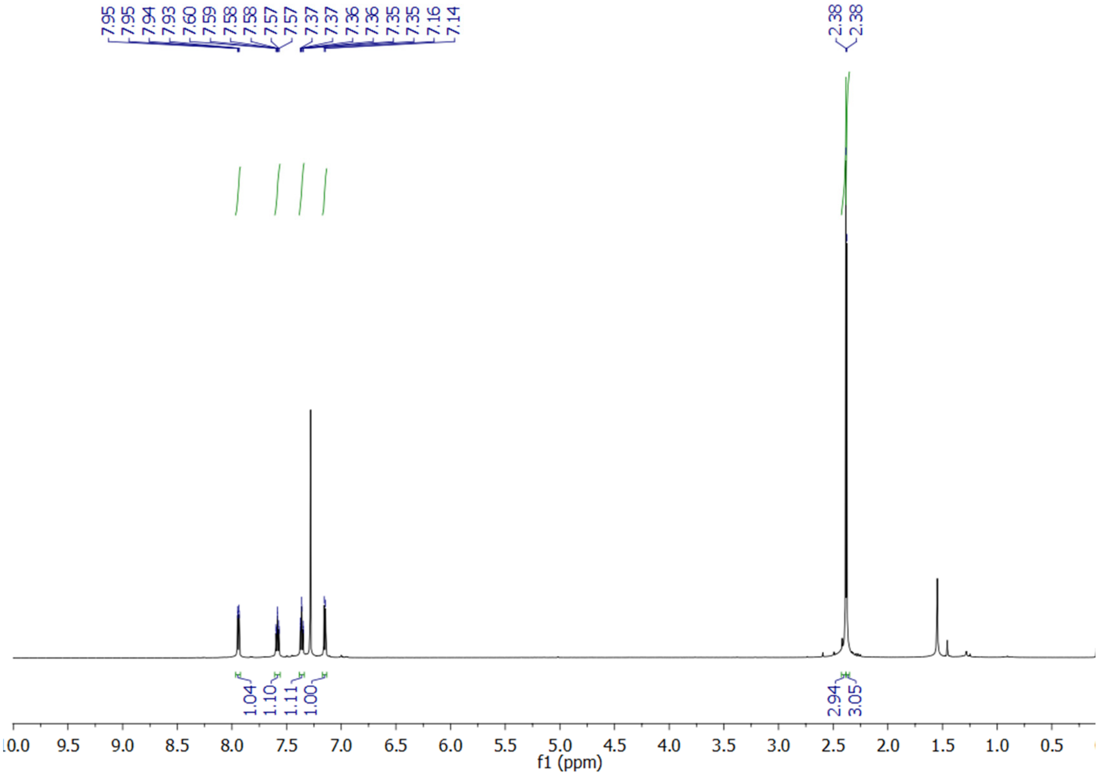

Figure S2. ^{1}H-NMR of **1a**.

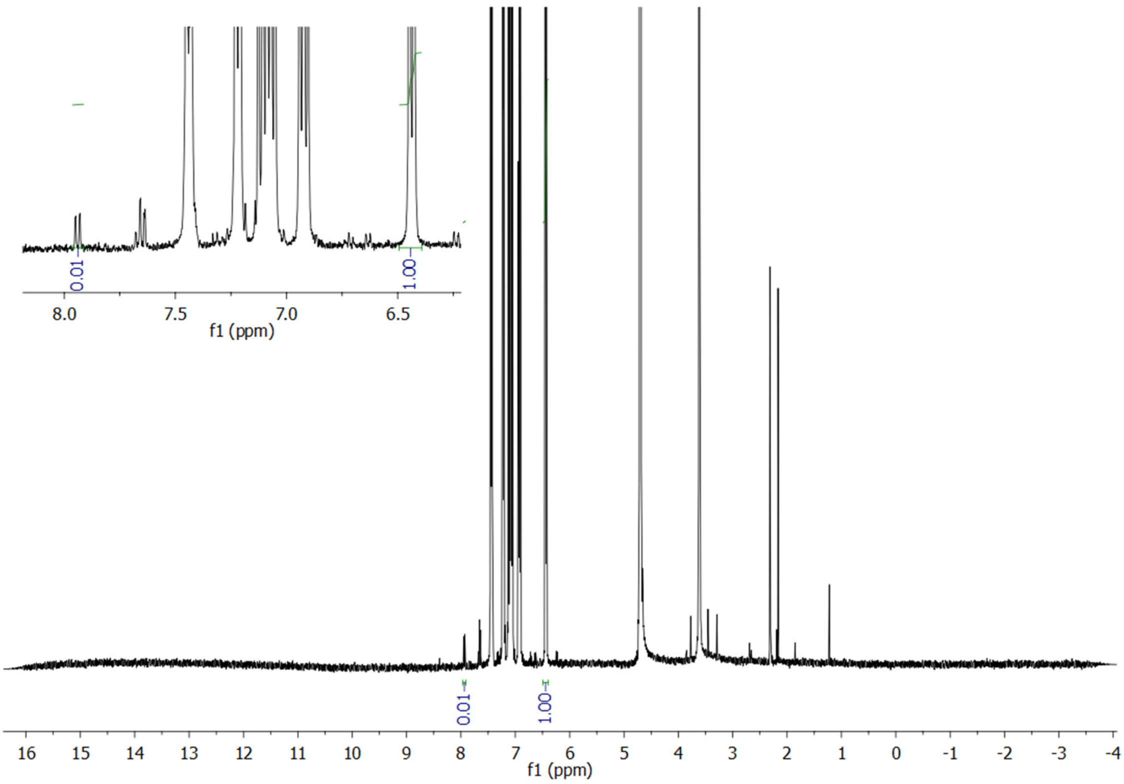

Figure S3. ^{1}H-NMR of **1a** and diclofenac sodium salt.

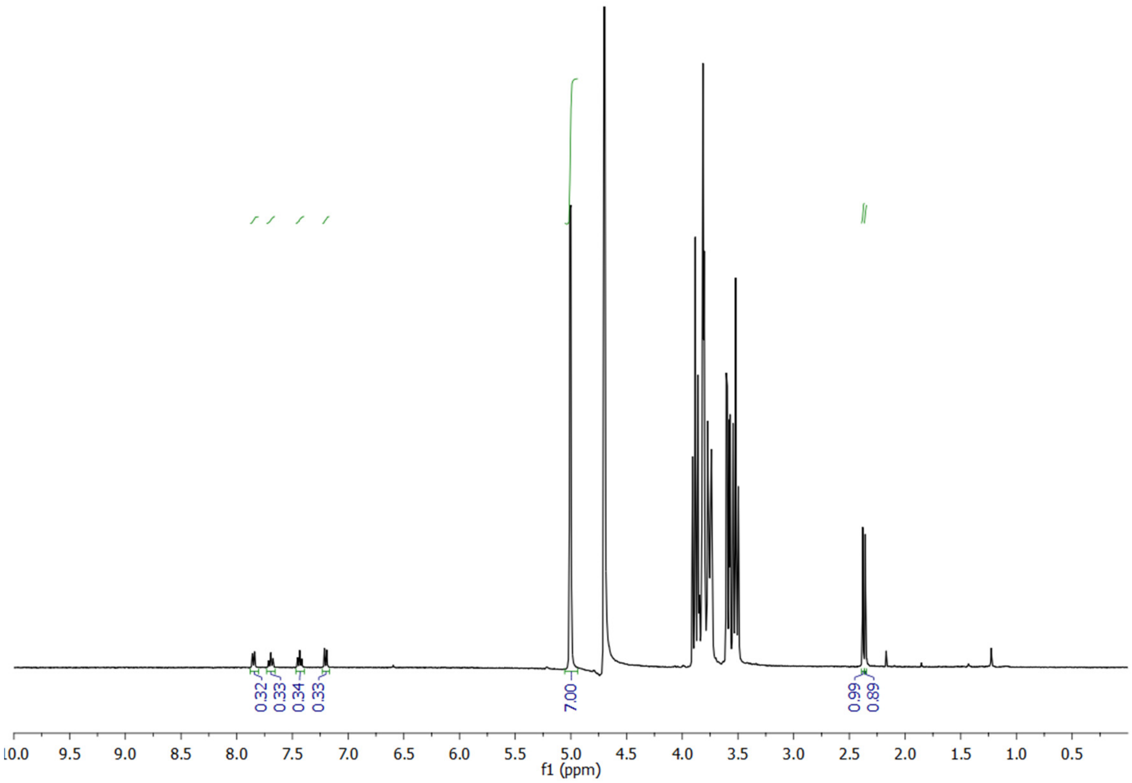

Figure S4. ^{1}H-NMR of **1a** and β-CD.

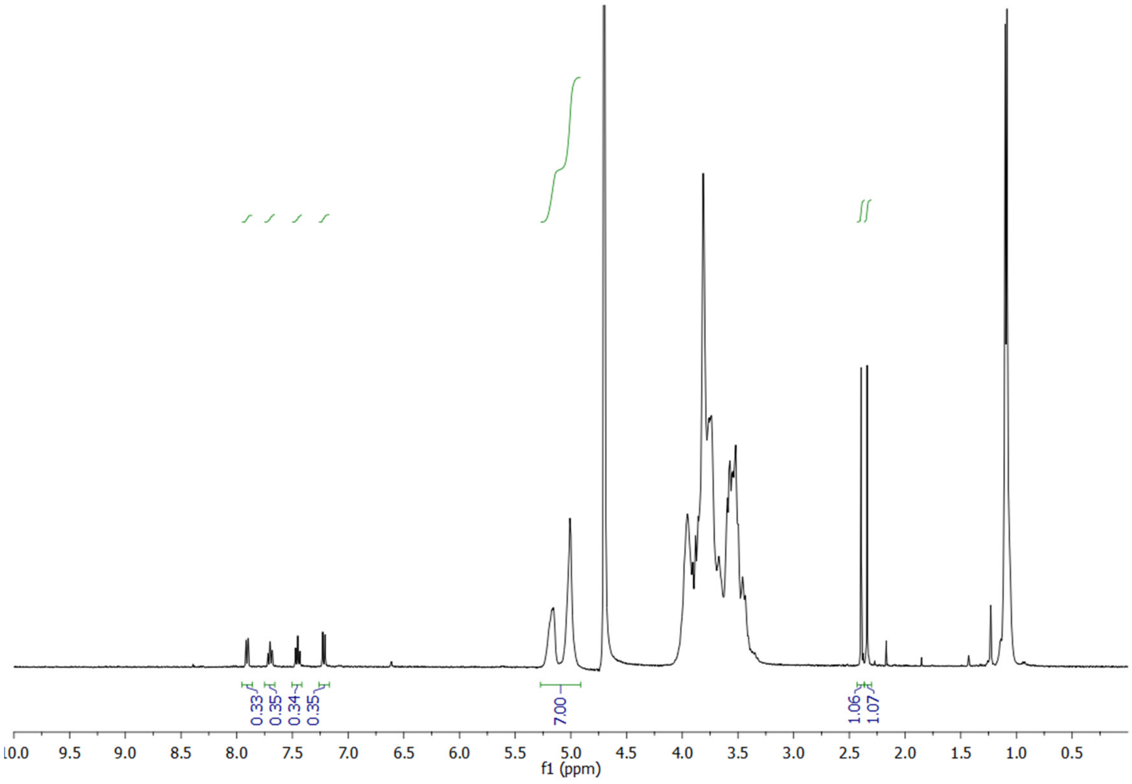

Figure S5. ^{1}H-NMR of **1a** and HP-β-CD.

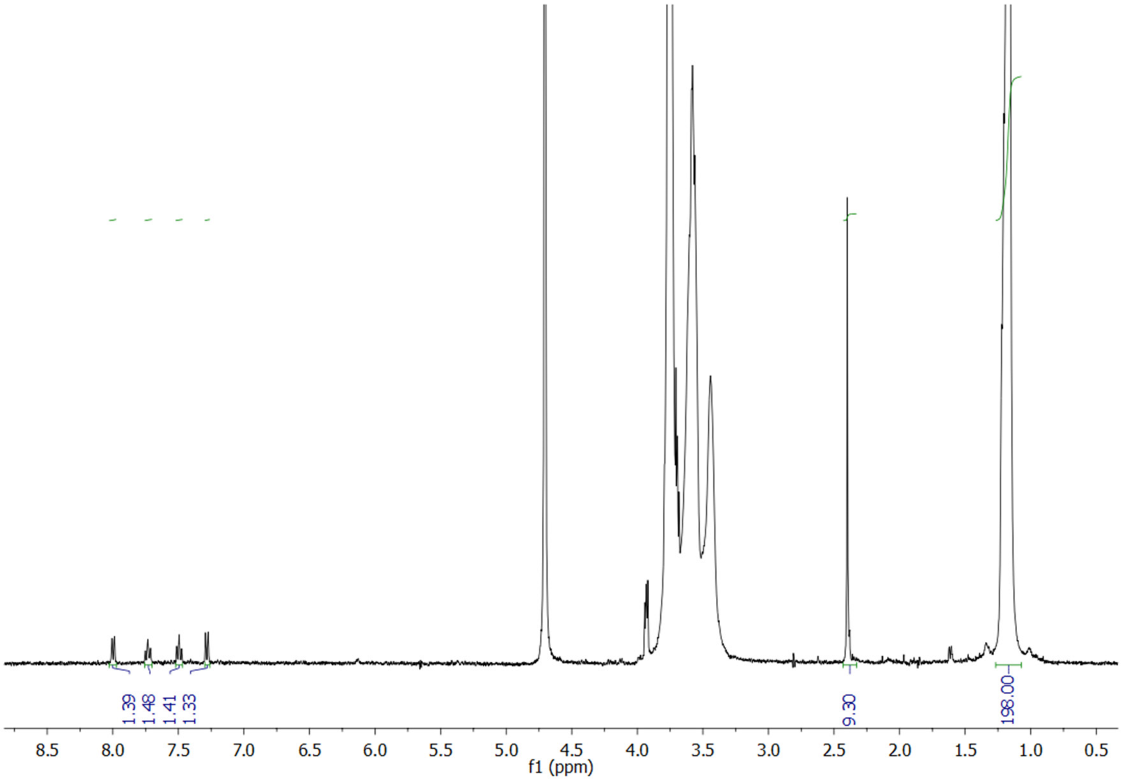

Figure S6. ^{1}H-NMR of **1a** and F127, at 37 ^{o}C.

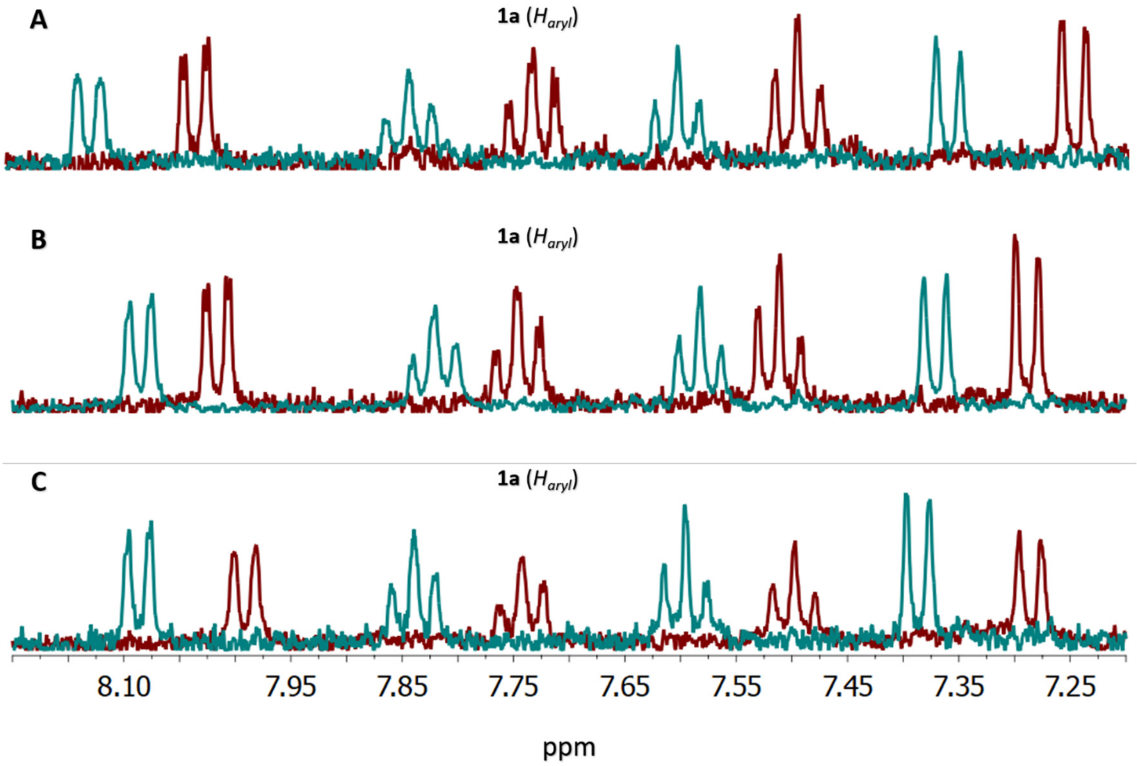

Figure S7. Expanded ^{1}H-NMR spectra of **1a** in the presence of Pluronic F127 (0.1, 1 and 5% (w/v)) showing the signal of aryl protons of **1a** (**A**, **B** and **C**, respectively), at 27 ^{o}C (red lines) and 37 ^{o}C (blue lines).
